# Supplementary material for: Synthesis, Physicochemical Characterization, Biological Evaluation, In Silico and Molecular Docking Studies of Pd(II) Complexes with P, S-Donor Ligands
Source: Pharmaceuticals (Basel). 2023 May 29;16(6):806. doi: 10.3390/ph16060806 (PMC10301315; doi:10.3390/ph16060806)
Supplement: Supplementary file 1 [file pharmaceuticals-16-00806-s001.zip › pharmaceuticals-2399393-supplementary.pdf]

Article

Synthesis, Physicochemical Characterization, Biological Evaluation, In Silico and Molecular Docking Studies of Pd(II) Complexes with P, S-Donor Ligands

Hizbullah Khan [1,*], Muhammad Sirajuddin [1,*], Amin Badshah [2], Sajjad Ahmad [3], Muhammad Bilal [4], Syed Muhammad Salman [5], Ian S. Butler [6], Tanveer A. Wani [7] and Seema Zargar [8]

[1] Department of Chemistry, University of Science and Technology, Bannu 28100, Pakistan
[2] Department of Chemistry, Quaid-i-Azam University, Islamabad 45320, Pakistan; aminbadshah@qau.edu.pk
[3] Department of Health and Biological Sciences, Abasyn University, Peshawar 25000, Pakistan;
sajjademman8@gmail.com
[4] Department of Chemistry, Kohat University of Science and Technology, Kohat 26000, Pakistan;
mbilal@kust.edu.pk
[5] Department of Chemistry, Islamia College University, Peshawar 25120, Pakistan; salman@icp.edu.pk
[6] Department of Chemistry, University of McGill, Montreal H3A 0B8, QC, Canada; ian.butler@mcgill.ca
[7] Department of Pharmaceutical Chemistry, College of Pharmacy, King Saud University, P.O. Box 2457,
Riyadh 11451, Saudi Arabia; twani@ksu.edu.sa
[8] Department of Biochemistry, College of Science, King Saud University, P.O. Box 22452, Riyadh 11451, Saudi Arabia; szargar@ksu.edu.sa
* Correspondence: dr.hizbullah@ustb.edu.pk (H.K.); dr.sirajuddin@ustb.edu.pk (M.S.)

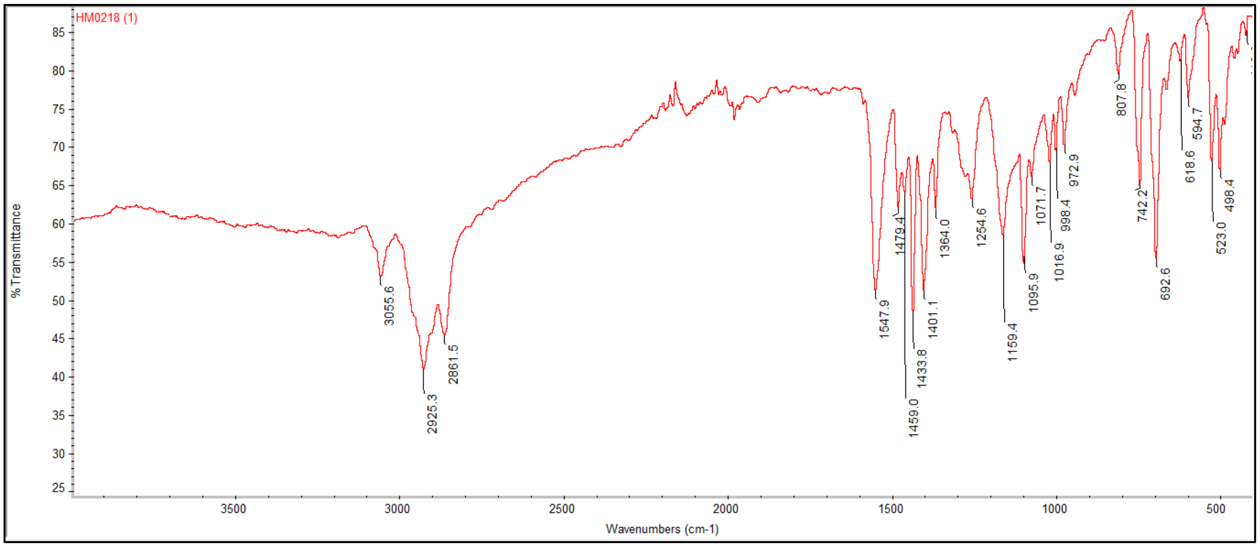

Figure S1. FTIR spectrum of complex **2**.

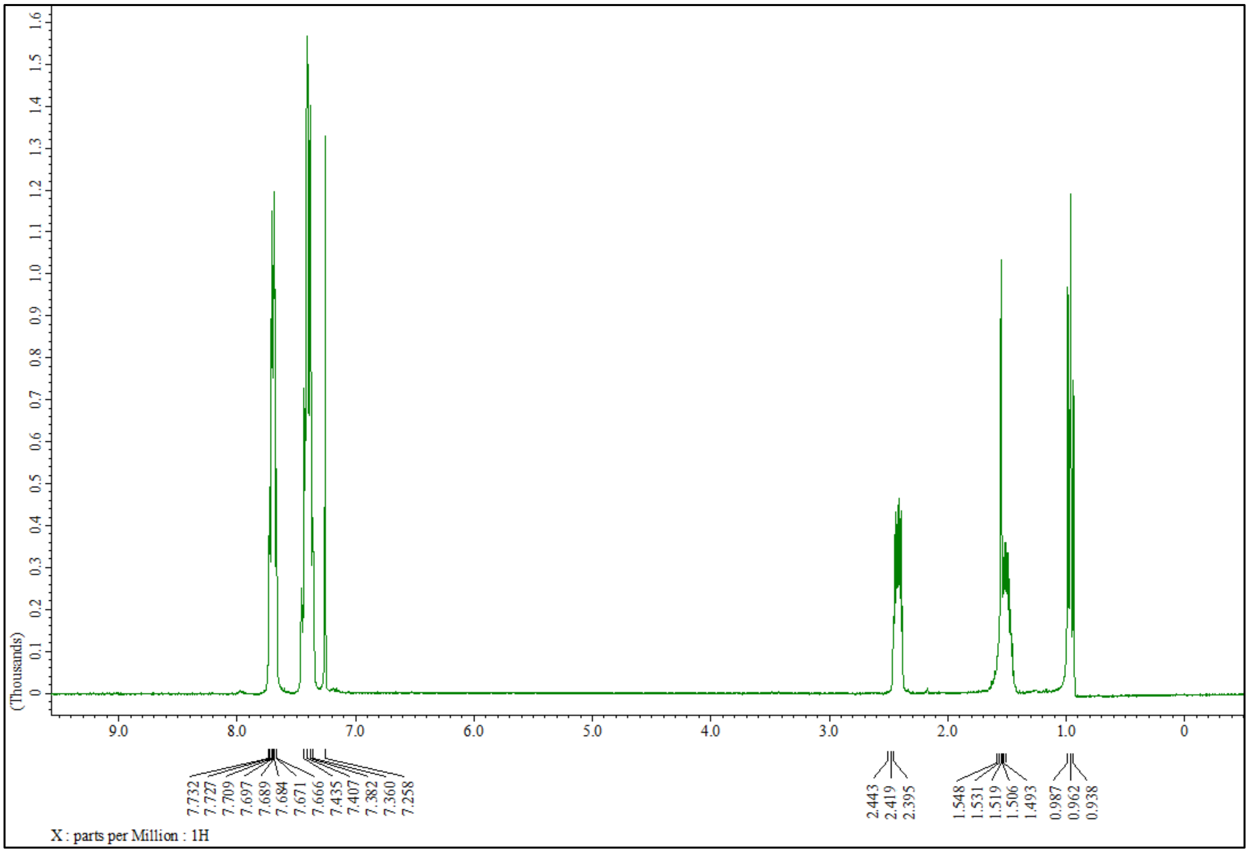

Figure S2. ^{1}H NMR of complex **1**.

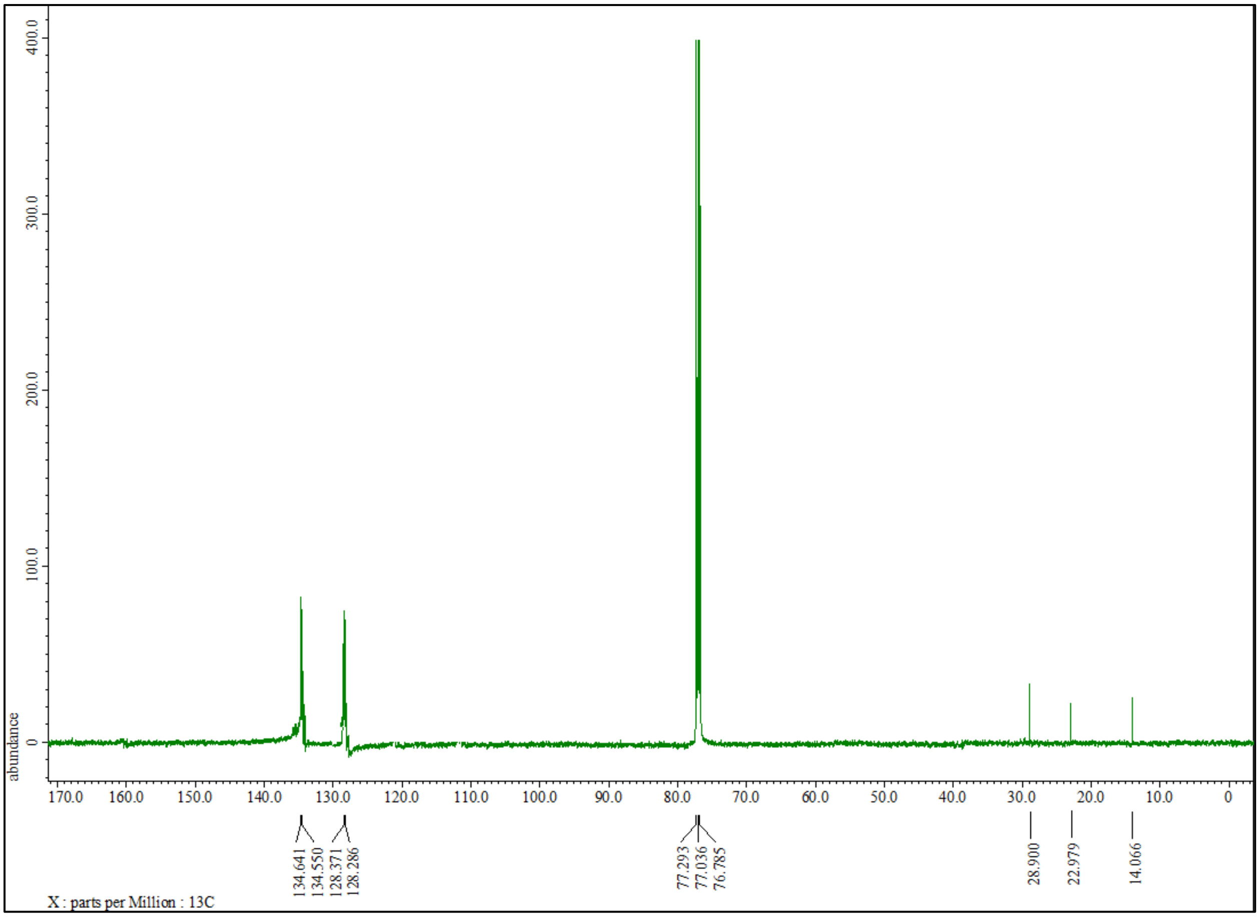

Figure S3. ^{13}C NMR of complex **1**.

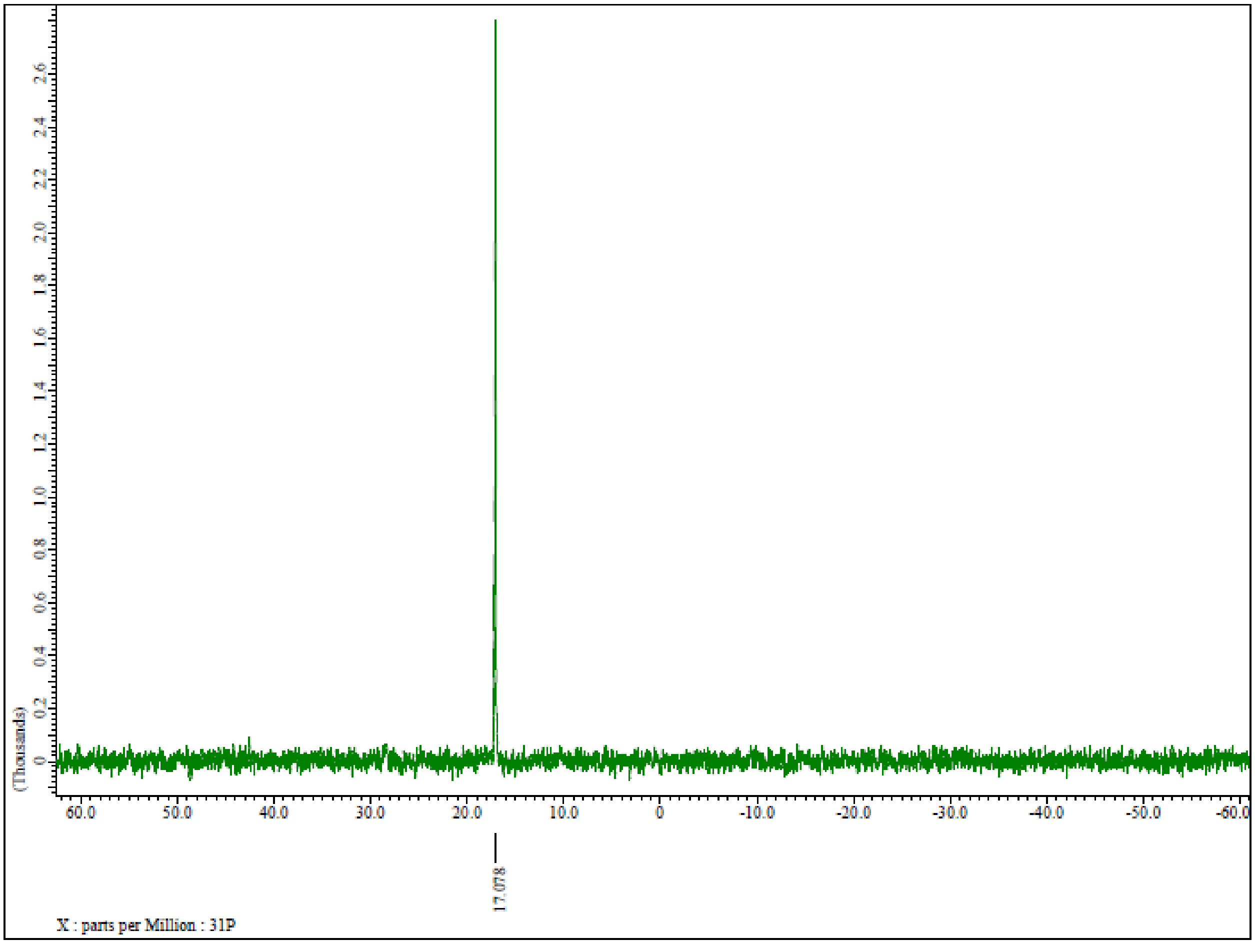

Figure S4. ^{31}P NMR of complex **1**.

Table S1. Binding energy score of starting organophosphorous Pd precursors with the receptor.

Receptor	Ligand	Binding Affinity (kcal/mol)
E. coli (PDB_ID: 6G9S)	potassium dimethylcarbamodithioate	-4.4988
	$(PR_3)_2PdCl_2$ where PR_3 = diphenyl-p-tolylphosphine	-6.3463
	$(PR_3)_2PdCl_2$ where PR_3 = diphenyl-2-ethoxyphenyl phosphine	-4.6034
	$(PR_3)_2PdCl_2$ where PR_3 = diphenyl-p-tolylphosphine	-6.8335
K. pneumonia (PDB_ID: 4EXS)	potassium dimethylcarbamodithioate	-4.0926
	$(PR_3)_2PdCl_2$ where PR_3 = diphenyl-p-tolylphosphine	-4.9375
	$(PR_3)_2PdCl_2$ where PR_3 = diphenyl-2-ethoxyphenyl phosphine	-5.3247
	$(PR_3)_2PdCl_2$ where PR_3 = diphenyl-p-tolylphosphine	-5.0306
S. aureus (PDB_ID: 5ZH8)	potassium dimethylcarbamodithioate	-4.3260
	$(PR_3)_2PdCl_2$ where PR_3 = diphenyl-p-tolylphosphine	-5.4957
	$(PR_3)_2PdCl_2$ where PR_3 = diphenyl-2-ethoxyphenyl phosphine	-6.1572
	$(PR_3)_2PdCl_2$ where PR_3 = diphenyl-p-tolylphosphine	-6.0522
DR5 (PDB_ID: 1DU3)	potassium dimethylcarbamodithioate	-4.3843
	$(PR_3)_2PdCl_2$ where PR_3 = diphenyl-p-tolylphosphine	-6.4459
	$(PR_3)_2PdCl_2$ where PR_3 = diphenyl-2-ethoxyphenyl phosphine	-7.1002

$(PR_3)_2PdCl_2$ where PR_3 = diphenyl-p-tolylphosphine	-6.9220

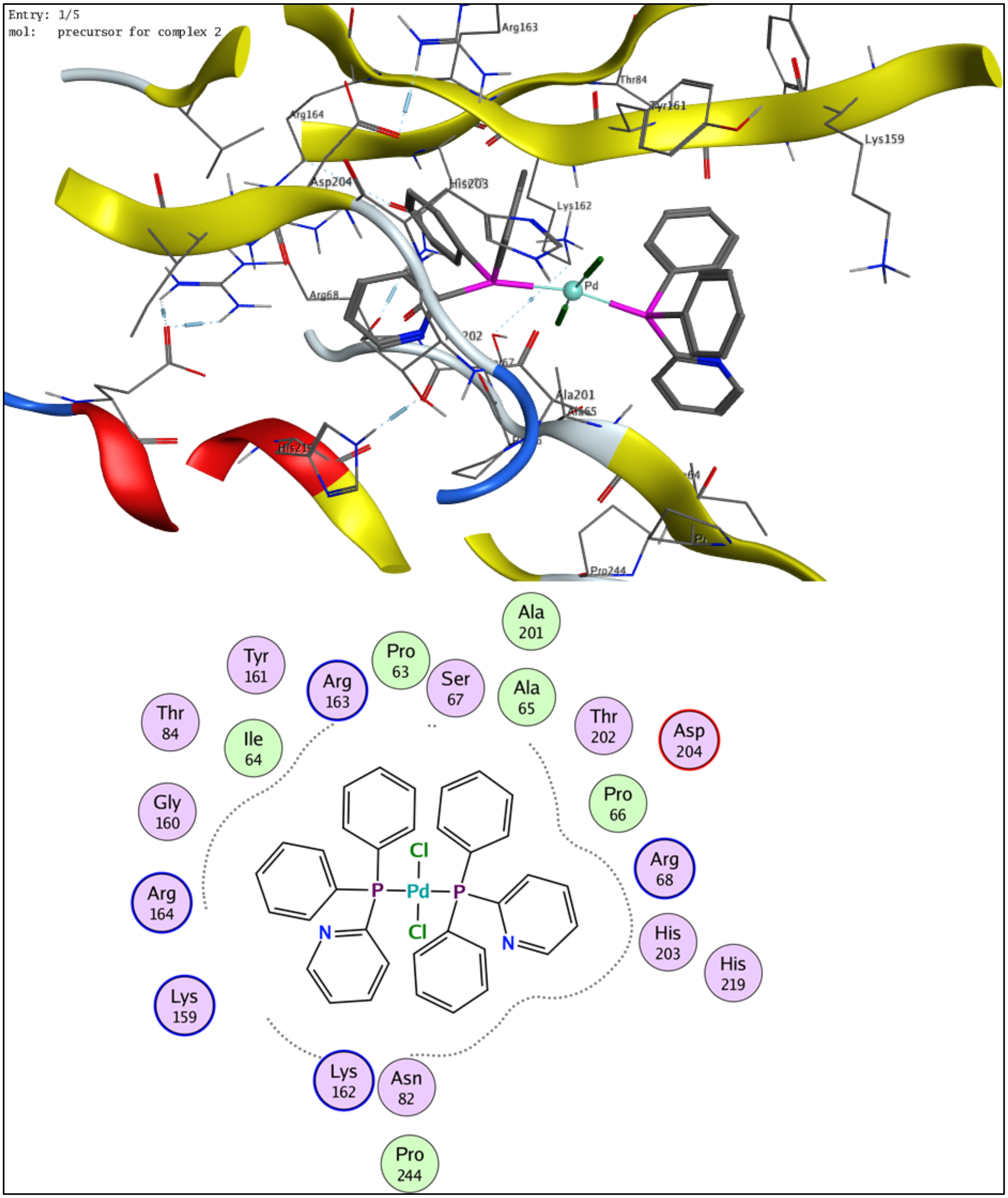

Figure S5. (A) Computed and (B) 2D interaction of $(PR_3)_2PdCl_2$ where PR_3 = diphenyl-p-tolylphosphine precursor with *E. coli* (PDB_ID: 6G9S).

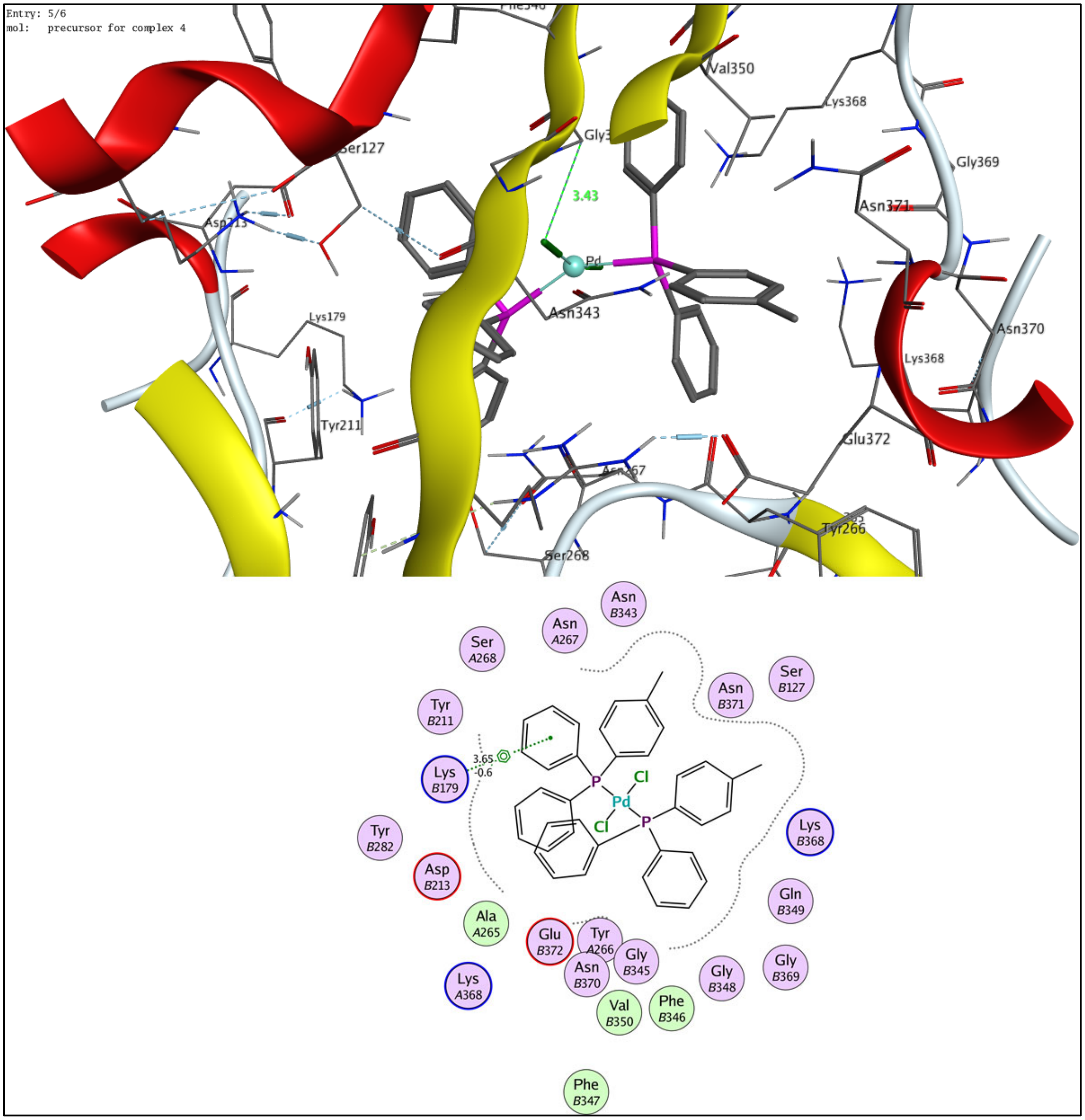

Figure S6. (A) Computed and (B) 2D interaction of $(PR_3)_2PdCl_2$ where PR_3 = diphenyl-p-tolylphosphine precursor with S. aureus (PDB_ID: 5ZH8)

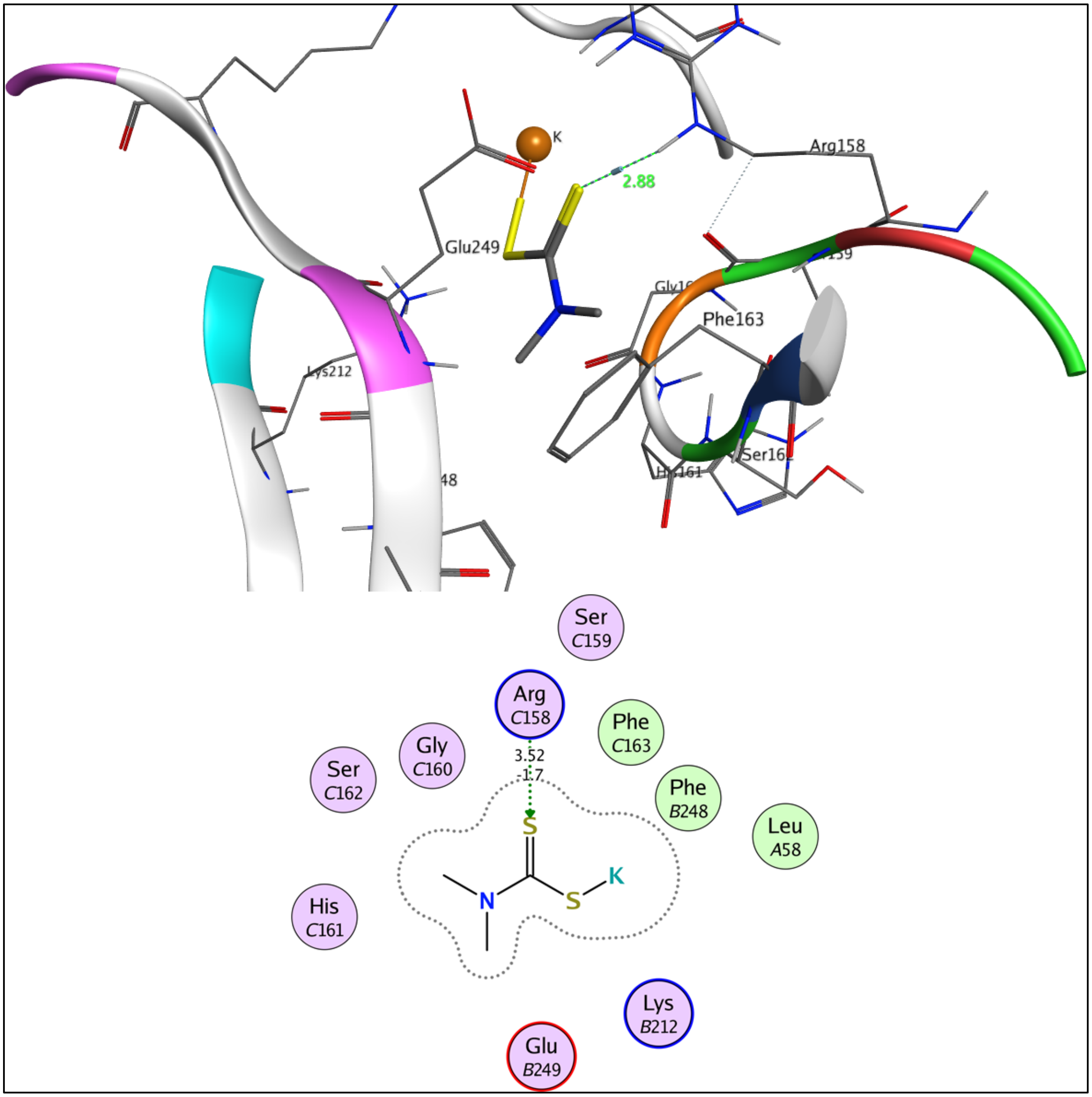

Figure S7. (A) Computed and (B) 2D interaction of potassium dimethylcarbamodithioate with DR5 (1DU3).

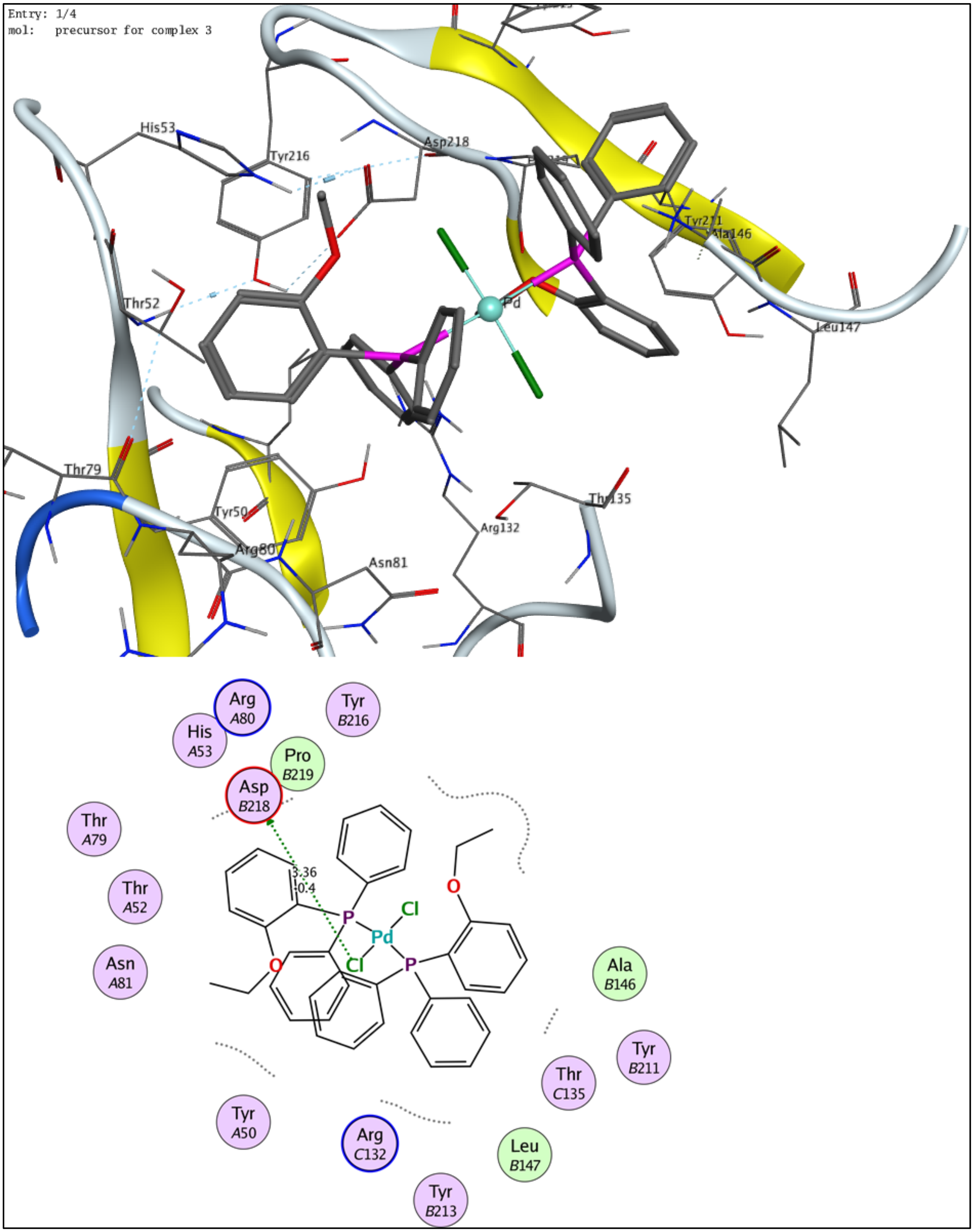

Figure S8. (A) Computed and (B) 2D interaction of $(PR_3)_2PdCl_2$ where PR_3 = diphenyl-p-tolylphosphine precursor with DR5 (1DU3).
